# What does receiving autism diagnosis in adulthood look like? Stakeholders’ experiences and inputs

**DOI:** 10.1186/s13033-023-00587-6

**Published:** 2023-06-08

**Authors:** Parisa Ghanouni, Liam Seaker

**Affiliations:** Department of Occupational Therapy, Dalhousie University, PO Box 15000, Halifax, NS B3H 4R2 Canada

**Keywords:** ASD, Late diagnosis, Adulthood, Experiences

## Abstract

**Introduction:**

The age of diagnosis is crucial for optimal health outcomes; however, some individuals with Autism Spectrum Disorder (ASD) may not be diagnosed until adulthood. Limited information is available about the lived experience of receiving a diagnosis during adulthood. Thus, we aimed to investigate stakeholders’ experiences about the ASD diagnosis during adulthood.

**Method:**

We interviewed 18 individuals including 13 adults with ASD who had received a late diagnosis during adulthood and 5 parents of individuals with ASD from various Canadian provinces.

**Results:**

Using a thematic analysis, three main themes emerged: (a) noticing differences and similarities, (b) hindering elements to diagnosis, and (c) emotional response to diagnostic odyssey.

**Conclusion:**

This study adds to the literature about experiences of receiving ASD diagnosis in adulthood. Given the impact of diagnosis on individuals, it is important to minimize the barriers to ensure individuals who require ASD-related supports can access them in a timely and effective manner. This study highlights the importance of receiving an ASD diagnosis and facilitates positive health outcomes. The findings from the current study can be used to guide adult diagnostic processes and practices to help make ASD diagnosis more accessible.

Autism Spectrum Disorder (ASD) is a life-long neurodevelopmental condition characterized by pervasive impairments in two main domains: social communication and social interaction as well as repetitive patterns of behaviors and interests [1]. Previous research demonstrates increases in the prevalence of ASD, however, underlying cause(s) for these increases are unknown [2]. Some postulate that this rise may be due to non-etiological factors such as changes in reporting practices, diagnostic criteria, or greater public awareness [2, 3]. The diagnostic criteria in DSM-5, saw the amalgamation of previously distinct subtypes of autism disorder into a single diagnostic category of ASD, as well as the reduction of core domains of impairment [4]. Currently, the best diagnostic methods involve an interdisciplinary team of healthcare professionals and should include standardized observations, medical and developmental history, assessments of the child’s learning and cognitive abilities, as well as interviews with parents and teachers who may be aware of “red-flags” [4–6]. The age of diagnosis is crucial for optimal outcomes as early intervention programs can lead to improvements in cognitive and language abilities as well as adaptive behaviour by capitalizing on the inherent neuroplasticity of a juvenile brain [7–11]. Earlier age of diagnosis has also been associated with reduced long-term societal and familial costs [10–12].

Despite the growing public awareness, lower costs, better outcomes with early intervention, and more reliable early diagnostic methods, many individuals are not diagnosed until they are of school age or older [13, 14]. This delay in diagnosis has the potential of postponing access to early interventions thereby decreasing their efficacity [7, 9, 10, 12]. There are a number of clinical features which have been associated with earlier age of diagnosis such as, increased severity of ASD symptoms, intellectual disability, language delays or regression, and history of medical problems related to ASD [7, 8, 10, 15]. In addition, research has demonstrated a number of non-clinical factors associated with earlier age of diagnosis, such as, higher parental education, greater parental concern, greater provider knowledge and experience related to ASD, having an older sibling, and geographical access to healthcare services [8, 10, 15].

Although research has demonstrated difficulties acquiring an ASD diagnosis during childhood, the process of obtaining an ASD diagnosis during adulthood presents unique challenges comparatively to childhood diagnosis [16]. Primarily, adulthood diagnosis may be more difficult as it requires that symptoms be present since childhood and therefor relies significantly upon medical and developmental history, which may be unobtainable or susceptible to recall bias [16, 17]. Furthermore, healthcare’ lack of ASD specific knowledge has consistently been shown to be a major barrier to diagnosis [16, 18–21]. Narrow and stereotyped views of ASD and its manifestation in adults lead to delays in diagnosis and poor referrals [19, 20, 22]. Additionally, the high costs of diagnostic services and the complexity of navigating the healthcare system affect the ability to access an official diagnosis [20, 22–24]. Health care providers cite systemic barriers such as a lack of funding, long wait-times, increases in referrals and caseloads, as well as limited access to resources as impacting their effective and timely services [18].

Many adults with ASD symptoms, and without official diagnosis, have noted that they would have preferred receiving a diagnosis earlier in life as it would have granted them access to services [19, 25]. Acquiring an official diagnosis gives individuals a framework to re-examine their past experiences, allowing them to reduce self-blame [26]. Individuals have also noted that their lack of understanding of their differences and challenges exacerbate existing mental health difficulties [21]. Additionally, an official diagnosis grants individuals with ASD access to a network and community of individuals like them, contributing to group identity and giving them a space to feel understood and accepted [26]. However, there are certain individuals who view receiving an ASD diagnosis as frustrating due to the lack of cure or clear prognosis [21]. Although ASD is being more researched, to our knowledge there currently exists a dearth of research investigating the subjective and lived experience of individuals receiving an ASD diagnosis during adulthood. Given the benefits of early diagnosis and the importance of diagnosis in addressing health risks, there is a need for further investigation regarding individuals’ experiences with diagnostic pathways. This study aims to investigate the subjective and lived experience of individuals diagnosed with ASD during adulthood from the perspectives of adults with ASD and their parents in an attempt to enhance the quality of services.

## Methods

The current study was qualitative in nature with the purpose of exploring stakeholders’ views pertaining to adulthood diagnosis of ASD. The primary research question investigated stakeholders’ lived experiences and perspectives regarding receiving an ASD diagnosis during adulthood.

### Sample

The current study recruited a population of 18 stakeholders, comprised of 13 individuals with high functioning ASD and 5 parents and/or guardians of individuals with ASD. Our participants were distributed across Canadian provinces including: 3 from Alberta, 2 from Quebec, 4 from Nova Scotia, 2 from Prince Edward Island, 2 from New Brunswick, 2 from Ontario, 1 from British Columbia, and 2 from Manitoba.

To meet the inclusion criteria, (a) individuals with high functioning ASD had to be diagnosed with ASD after the age of 18 as to be considered a late diagnosis, and (b) parents and/or guardians of individuals with ASD, had to have an adult child with ASD. In this project, late diagnosis was operationally defined as 18 years or older, which is linked to an age after adolescence [27]. Given that the average age of ASD diagnosis in Canada is before age of four years old, the diagnosis at 18 years old or older could provide an excellent base to help uncover contributing factors for such a late diagnosis. All participants had to be able to verbally communicate in English to be eligible for participation.

Adults with ASD ranged in age from 27 to 53 years and all lived in urban areas. Additionally, 10 adults with ASD that participated had one or more co-occurring diagnoses: six had an ADHD diagnosis, three had learning disabilities, six had mental health disorder, and one had a metabolic disorder. Individuals received their official ASD diagnosis between the ages of 18 and 52. Parents of adults with ASD ranged in age from 46 to 63 years and all had one adult male child with ASD. Three parents lived in rural areas and two lived in an urban area. They reported that their adult children had one additional comorbidity including: one with ADHD, two with learning disability, and two with other mental health conditions. Their adult children ranged in age from 25 to 36 [mean (SD): 29.75 (4.57)] and were officially diagnosed between the ages of 18 and 36 (See Table 1).

**Table 1:** Demographic information of participants

Participant group	Number	Sex: male/female	Age: mean (SD)
Individuals with ASD	13	9/4	40 (8.44)
Parents	5	2/3	58 (8.04)

### Recruitment

Using convenience sampling, we recruited participants by disseminating invitation letters and posters among networks of community centers, public and private clinics, and organizations related to ASD across Canada. We also used snowball sampling by asking interested participants to share the project information among their personal networks to target additional stakeholders. We continued recruitments until saturations in data, which is having no new concept in data, was reached.

### Data collection

Individuals who expressed interest were sent a consent form to sign. Then a 60-min interview was scheduled for a time and place that was convenient for participants. Individuals were also given the option to conduct the interview face to face, over the phone, or over Skype. The interviews were in-depth and semi-structured using open-ended questions and were audio-recorded using a password-protected recorder. Although our participants with ASD were high functioning with respect to social interactions, we asked them interview questions slowly and/or rephrased them when needed to better accommodate them. Participants were given pseudonyms to ensure anonymity and protect confidentiality. After having completed the interview, interviewees were sent a demographic survey to gather personal information such as age, gender, medical history, etc.

Although there is a strong argument for either identity first language or person first language, our participants in this study introduced themselves as "people with ASD" in the interviews and during the data collection. Following their choice, we used this term as per their preference. The current study has been approved by the University Behavioral Research Ethics board and all participants gave their consents prior to the study.

### Data analysis

Audio-recorded files were transcribed verbatim and then double checked for any errors by one research assistant. Researchers used constant comparative method to help increase the traceability and the credibility of their analyses [28]. We used conceptual framework of Access to Health during the analysis [29]. This framework proposes several dimensions of access and helps evaluate the complex and dynamic process involved in the healthcare system and among communities. During the data analysis, two research assistants independently read to code transcripts and identify patterns using thematic analysis [30]. Then, the research assistants compared their codes to check for consistency, any disagreements were resolved using a meeting with a third team member. NVIVO software was used to analyze the transcripts, creating codes, categories, and over-arching themes [30].

To enhance credibility of findings, we used several strategies. We employed reflexivity using memos to ensure that our assumptions were not impacting the data analysis. Furthermore, our research team had several meetings prior to the data analysis to share their assumptions about the late diagnosis of ASD and how their backgrounds might influence the way they interpret the data. Additionally, having team members from different backgrounds allowed us to use triangulation and incorporate the views and perspectives of multiple researchers, which helped enhance the credibility of analysis.

## Results

The information gathered during the interviews with participants yielded three themes with regards to their subjective and lived experience of adulthood ASD diagnosis: (a) noticing differences and similarities; (b) hindering elements to diagnosis; and (c) emotional response to diagnostic odyssey. Each theme encapsulate several concepts as illustrated in Table 2.

**Table 2:** The themes and concepts around late diagnosis

Noticing differences and similarities	Hindering elements to diagnosis	Emotional response to diagnostic odyssey
Public awareness	Cost of care	Emotional validation
Self-matching behaviours	Waitlists and wait-times	New perspectives about identity
Noticing a need	Lack of ASD specific knowledge	Regret and sorrow

### Noticing differences and similarities

Prior to seeking out official diagnosis, typically a circle of family or friends including guardians, teachers, or service providers may notice red-flags. Although observing differences and/or suspecting symptoms cannot always lead to receiving the diagnosis, stakeholders indicated that public awareness, self-matching behaviours, and noticing a special need can play a significant role in the diagnostic journey.

### Public awareness

Awareness about self and health conditions have been raised by our participants as one of the critical aspects to seek medical care. Many stakeholders noted a relative lack of information pertaining to ASD at the time of their childhood, leading to a late diagnosis due to missed red-flags. For example, Sebastian, an adult with ASD, explained his parents’ struggle to answer their suspicions: “You know, my parents had searched when I was a child, they had searched for answers [to my differences] and there were no answers in the 70’s.” Likewise, Lucy, an adult with ASD, noted: “Well… as an adult… when I was a kid a lot of this [autism symptoms] was not understood.” Jack, an adult with ASD, was able to recall the story of how his mother first learned about ASD at a dinner party:

“Actually, my mom um, noticed, like odd behaviors I had, and she went to a dinner party and…one of the guests there… said to her, 'Your son has Asperger’s syndrome' and she said, 'What, what's that? I don't know' and so he told her… Nobody knew about it in 91…so we read up on it.”

For many in the current population, the absence of red-flags led to a late-diagnosis.

### Self-matching behaviours

Many participants noted that reflecting on the similarities of behaviours between themselves and those diagnosed with ASD led them to begin their diagnostic journey. One of the common pathways for stakeholders was self-matching personal behaviours with individuals close to them who have received an ASD diagnosis. Some participants expressed that their concerns of ASD were validated during their relatives’ diagnostic procedure. Sebastian, an adult with ASD, noted: “even though I knew for seven years that I had to be autistic… since my sons’ diagnosis, [I learned] that I must have been autistic.” For others this validation was through their friends’ diagnostic journeys. For example, Sophy, an adult with ASD, said:

“It was only when, she [my friend] was diagnosed, and she explained [symptoms], and I asked her about her diagnoses, what happened and when she explained this to me, I told her, well, it sounds a lot like me when I was a child, and when I grew up.”

Some participants, such as Lily, an adult with ASD, who held suspicions of ASD prior to her diagnosis, were able to access their own diagnostic procedures through their relatives’ therapists. Lily noted: “After her [relative’s] assessment going in and talking to her therapist, um, she said do you think you might be too, and I said definitely am, there’s like no doubt about it. And then I think I asked her if I could be assessed.” These quotes help showcase how certain individuals begin their paths towards diagnosis through matching their behaviour with individuals close to them who have been diagnosed with ASD**.** These quotes highlight how noticing similarities in behaviours may be precursors for seeking out diagnosis.

### Noticing a need

In addition to seeking out diagnosis because of similarities of behaviours, individuals also sought out diagnosis because of difficulties associated with ASD. Milo, an adult with ASD, described his experience of diagnosis due to exacerbated symptoms and sensory issues:

“I was diagnosed with autism around the age of 16 and 17. Things weren't going particularly well, well at all. Starting around the age 13, 14, I started having um really bad phobia, really bad sensitivity to sounds, very debilitating… very hard. I was yelling at my mom and my dad for making all these noises with their mouth.”

This shows how challenges associated with ASD may be informative for seeking a formal diagnosis. Although most participants began their diagnostic journeys via conventional routes of seeking help from service providers, some participants discussed gaining access to diagnosis through school. Maria, a parent, commented on this: “There’s a lot of people who are diagnosed through, you know through a behavioral challenge in school.” This shows that behavioural challenges and the need for special services in the school may be an avenue to seek diagnosis. Similarly, Cameron, an adult with ASD, explained how his diagnosis came along due to difficulties at school: “Mainly that [diagnosis] came about because I failed grade 1, I think, and then, I got diagnosed and just got help with it through like the school.” However, this is not always the case as William, an adult with ASD, explained: “ [when] I finally got diagnosed with autism [as an adult], the psychologist said, I should have been diagnosed with autism when I was kid. She looked at all my reports, she said it was very clear that I had, but it wasn't clear to the people in school.” These quotes from participants suggest alternative pathways by which individuals can find differences in their functioning that lead to seeking medical diagnosis.

### Hindering elements to diagnosis

Once individuals begin their diagnostic journeys, they are often faced with challenges which hinder their ability to access diagnosis in an effective and timely manner. Our stakeholders described several elements, including cost of care, waitlists and wait-times, and lack of ASD specific knowledge in service providers, which affected the accessibility and the quality of diagnosis and subsequently impacted their ability to receive diagnosis on time.

### Cost of care

Once individuals can find diagnosis services which are offered, the next step is being able to afford them. Participants commented on the high cost of care which hinders their ability to access diagnostic services. Nora, a parent of an adult with ASD, explained what it cost her to receive diagnosis: “I had to lose my career, my life savings, and my home in order to get a proper diagnosis.” Suzy, an adult with ASD, shared similar sentiments and elaborated on her difficulties:

“It was a big struggle for me finically because like many autistic people, I was under employed and not well paid. So, I'm like always living from paycheck-to-paycheck hand to mouth basically. So, I have no means of saving any money, and it costs 1000 plus dollars for this diagnosis, so I had hopes that maybe one day I could save up for it in 5years or something.

Similarly, Laura, an adult with ASD said: “I could only afford, four other sessions that was it- at 200 dollars an hour its way too expensive for people like myself, to try to figure how we fit in the world.” These quotes showcase the financial burden that accompanies diagnosis and highlight the need for more affordable care.

### Waitlists and wait-times

In addition to the high costs of care and the time it can take to save up for diagnosis, participants also commented on the long waitlists and wait-times when accessing services. Sebastian, an adult with ASD, commented on the length of time it took to access diagnosis: “One of the most frustrating things that ever happened…, we waited forever to get the top of the waiting list to get services.” In the case of some individuals, the wait times were even unknown. Suzy, an adult with ASD said: “I was put on a waiting list, and they couldn't tell me how long the waiting list will be.” However, this is not always the case, as some individuals were able to access diagnosis with the support of others. Madison, a parent of an adult with ASD, explained the support of her sister to access diagnostic services early: **“**Well, I'm very unique, I had a sister that worked in the hospital system. and got him into a doctor really quickly…, So, I was very blessed”.

The quotes gathered from participants expressed a need for expedited diagnostic services to ensure that individuals receive timely diagnosis regardless of age.

### Lack of ASD specific knowledge

In addition to facing barriers in the process of accessing diagnosis, participants commented on experiencing challenges related to the quality of care. Participants noted a lack of ASD specific knowledge in healthcare settings. A sentiment expressed by Sophy, an adult with ASD: “I wish that health professionals in general, all over, were better informed.” For some participants the lack of healthcare provider ASD specific knowledge led to misdiagnosis as well as mistreatment, as Drake, an adult with ASD, noted: “make sure your diagnosis is accurate [laughs], I'll say that right now because most of my life I was diagnoses with ADD so I was getting the wrong treatment and medicine, so, it was a long till I was diagnosed with autism.” Tara, an adult with ASD commented on the lack of knowledge of ASD and listening to clients’ complaints that can affect the care outcomes. She said: “I find a lot of times, if you have something like autism, even then you go to talk to them [service providers] about a different concern, they always think that it has to do, it’s because you’re autistic. But that’s not always true. So, it takes a long time for them to finally listen.” Lily, an adult with ASD, recalled her long-term healthcare struggles and how she could not find an appropriate management strategy:

“I've always struggled with depression, and I've always been trying to figure out this, like it was depression but it wasn't depression. I couldn't understand how I could always feel depressed but nothing, but nothing depression related was working. …. I could never understand why until 44 and I got diagnosed [with ASD].”

### Emotional response to diagnostic odyssey:

When individuals receive their diagnosis a range of responses is typical. Most notably individuals experienced an emotional validation, new perspective and understanding surrounding their identity, and feeling of regret after the diagnosis.

### Emotional validation

As a result of receiving late diagnoses and living without services for a long duration, many participants experienced hardships in their life. Receiving a diagnosis and gaining a better understanding of their challenges proved to be an incredibly validating experience for participants. Sebastian, an adult with ASD, expressed the emotional magnitude of receiving his diagnosis:

“When I finally got that diagnosis that validation was earth shattering… because having that information is powerful and validating, you know? And it gives you a vocabulary, you understand why and how things are happening… it was amazing. I cried for three days… because I was … finally validated, I barely slept for three days because I was just shaking and crying with the relief of finding out that I was right after all.”

Laura an adult with ASD indicated her emotion after receiving diagnosis and said: “I literally cried from joy”. Suzy, an adult with ASD, shared similar sentiments: “I just broke down sobbing from relief and joy that all my struggles are validated… it was a huge thing for me, this diagnosis and I was so overjoyed to finally know why I struggled all my life, and so many things made sense.” These quotes highlight the emotional validation that individuals receive from the new lens and outlook diagnosis offers them.

### New perspectives about identity

Receiving diagnosis helped individuals develop a better understanding about themselves. Sophy, an adult with ASD, explained: “I never knew what was the matter with, until I’m 63, I just knew that somehow, I always was different, but now I understand much better what was the matter with, what is the matter with me.” For some participants, the understanding acquired from the opportunity to re-examine past experiences via a new lens fostered a stronger notion of self. Sebastian, an adult with ASD, explained how the diagnosis impacted his notion of self:

“So, for me diagnosis was a game changer, it really was a paradigm shift in my thinking about myself... it was, it was like a thunderclap. Getting that diagnoses just was so profoundly changing for me because there was a lot of emotional baggage that I could put down… I wasn't a bad person; I was autistic…, and that really made a change, … I'm not a freaky neuro-typical, I'm a perfectly normal autistic and that’s okay that I'm different.”

Participants also expressed how receiving diagnosis impacted their life choices moving forward. Jack, an adult with ASD, explained: “Well, it [diagnosis] helped me because I didn't know what I had or anything growing up.” Lily, an adult with ASD, expanded on that idea:

“Since my diagnosis I've re-evaluated how [my life is], I'm trying to re-vamp my entire life so that I'm making accommodations for myself knowing what I know now. Um, so I would want to know, when I'm going into a place where I hope to be working, I would want to know that it was autism friendly I guess... I would want to know that I wasn't facing barriers in communication.”

For many participants receiving an official diagnosis granted them a new perspective to view themselves about past experiences and plan for the future.

### Regret and sorrow

Although receiving diagnosis was overall a positive event for individuals, participants expressed sorrowing over post-diagnosis. A common frustration conveyed by participants was the opportunities missed due to the late diagnosis. Sophy, an adult with ASD, commented on the difficulties she experienced due to her lack of diagnosis: “And it’s a pity I didn't know at earlier age, and that the adults surrounding me didn't know.” Lily, an adult with ASD, noted how different her life may have been with an earlier diagnosis: “If I'd had known as a kid, I mean I couldn't even imagine. Like it just would have changed so much.” Jack, an adult with ASD, expanded on the benefits of such a wish:

“You know, they [others] get diagnosed as early as possible so they [service providers] can help them to have them a most meaningful and fulfilling life that they can, and it's be easier on the child and the parent, everybody I think. It just helps everybody I think, if they can get as much help as they can early on.”

Sebastian, an adult with ASD, commented on the continued life difficulties after diagnosis due to the lack of resources. He said: “I would always want to live as a diagnosed person where I have the control to live, the life that is hurting me the least, um because life is still going to be hard [after the diagnosis].” Sebastian, expanded on this idea commenting on the lack of supports in many areas of daily life, saying:

“After having had the diagnoses which changed things for me emotionally and mentally and you know having accommodations and finally getting good grades in my life, you know? I still failed at work, I still failed because the world isn't set up for me…not having supports and accommodations, it just broke me.”

These quotes illustrate the feeling of regret that individuals feel due to receiving diagnosis so late in life, highlighting the need for more timely diagnosis.

## Discussion

This study explored the subjective and lived experience of receiving an ASD diagnosis during adulthood, through the perspectives of adults with ASD and their parents, with the aim of enhancing the quality of services available to individuals with ASD. Although there is growing literature pertaining to the experience of receiving an ASD diagnosis in childhood, to our knowledge few studies have focused on the subjective experiences of individuals receiving a late ASD diagnosis. This study highlights the different ways by which individuals come to seek diagnosis, the need for better and more accessible services, as well as the emotional odyssey of being diagnosed later in life.

Although research has indicated a global increase in public awareness pertaining to ASD, variability in knowledge as well as misconceptions regarding ASD’s symptomology and presentation remain. The different levels of knowledge among the public may impact how and when “red-flags” are noticed as well as the potential ways they use to seek out diagnosis [18, 31, 32]. Our participants noted that ASD public awareness was not high earlier in their lifetimes, which led to stakeholders missing red-flags. In addition to the regular professional check ups and screening, circles of families and friends can recognize warning signs at early stages. Given that individuals with ASD spend most of their developmental time with parents and educators, they play a unique and essential role in identifying developmental and behavioural concerns as well as noticing individual’s special needs at an early age [33]. Thus, it is assumed that increasing awareness among all stakeholders, primarily teachers and parents, can help facilitate the detection of warning signs and decrease the age of receiving professional assistance [34].

The increased awareness and understanding of ASDs symptoms can help reinforce individuals’ health literacy to make appropriate health decisions [35–37]. Although a plethora of research has been conducted on health literacy in general, to our knowledge few studies have focused on how health literacy affects ASD diagnosis [36]. Research on health literacy suggests that the conceptual models individuals use to explain illnesses help shape their help-seeking behaviours as well as impact the choice of and the compliance with treatments [35, 37, 38]. To deal with poor health literacy, our participants stated that self-matching behaviours with individuals diagnosed with ASD was a common stimulus for beginning their diagnostic journey. Although self-matching behaviour is dependent on having someone with ASD within individual’s circle of family/friends and it appears to be a common path towards diagnosis for individuals lacking proper health literacy, it may not provide comprehensive information about symptoms. Increasing individuals’ and their families’ health literacy could help them access diagnostic services in a timely manner Given the importance of individual’s capacity to understand and access information, it is critical to develop family’s and individual’s health literacy to ameliorate health outcomes as well as decreased health inequalities [35, 39].

Once adults with ASD begin their diagnostic journeys, they may experience a multitude of barriers in accessing diagnostic services. Aligned with previous literature, the high cost of diagnostic services can be a major barrier [16, 24]. Adults with ASD may have left the financial umbrella of their parents and missed the opportunity of receiving childhood financial services/support. Financing care for adults with ASD may be particularly difficult as individuals with ASD frequently have difficulty finding and maintaining employment [40, 41]. Additionally, the long waitlists and wait times experienced by participants in the current study affected individuals’ ability to access diagnostic services [16]. Previous research has found that the number of services offered to individuals after childhood often drop and individuals struggles to find appropriate resources [16, 18, 19, 24, 41]. This is in line with the findings from the current study as many participants expressed difficulties finding appropriate diagnostic services during adulthood as well as professional services offered after the diagnosis.

Findings from the current study suggest that even once individuals are able to access diagnostic services, a lack of specific knowledge about ASD in adulthood amongst professionals may impact the quality of care. Potential narrow and stereotyped views regarding ASD among service providers may affect timely and accurate diagnosis [16, 19, 42]. It has been suggested that healthcare professionals can benefit from taking more ASD-related training to help them with accurate differential diagnosis in adults with ASD as many autistic traits may have comorbidities with other mental illnesses [16, 43]. Additionally, further training may help providers ameliorate their interactions with patients, a key factor in a positive diagnostic experience [44, 45]. Actively listening to patients and using alternative communication methods to involve them in decision making process may help improve the quality of communication [45]. Research has noted that individuals with high-functioning ASD may be at a higher risk of being missed due to not fitting the stereotypical presentation of ASD and using camouflaging techniques [44, 46]. This can lead to mental health difficulties being overlooked or assumed to be part of ASD symptomology, which often limits access to timely resources [18].

Individuals in the current study viewed receiving an ASD diagnosis mostly as a positive experience, fostered by the ability to re-examine painful past life experiences through a new ASD framework. Such emotional validation can help individuals reduce any feelings of blame or shame they felt from social misunderstandings by shifting some responsibility of difficult past experiences to the diagnosis [21, 26, 46]. However, our participants expressed feelings of regret and frustration caused by having to have lived their lives without a diagnosis or proper support, with many participants expressing their desire to have received diagnosis earlier in life. Additionally, our participants were frustrated by the availability and the quality of post-diagnostic services. Research has found that individuals receiving diagnosis in adulthood are often left without adequate formal direction towards seeking support post-diagnosis, leaving them with feelings of loneliness and abandonment as well as uncertainty regarding their futures [19, 41]. Leedham and colleagues highlighted the exhaustion and pain individuals experienced to be able to adjust at a late stage, with many participants showcasing feelings of grief caused by losing their past life and understanding of self [46]. Although literature examining childhood diagnosis have shown parental feelings of denial post-diagnosis, no such feelings were showcased in the adult population, indicating a welcomeness to the diagnosis [47]. This finding is novel and may indicate a readiness to accept diagnosis as well as the perceived benefits that accompany it after a life of difficulties and struggle without appropriate services.

Our participants stated that diagnosis permitted them to develop a renewed and better understanding of themselves and to strengthen their notion of self. This is in line with prior research, which has indicated that diagnosis can provide a sense of permission to further begin developing ones identity [46]. Individuals, who positively associate themselves with ASD or view ASD as a form of neurodiversity, often experience higher self-esteem and self-acceptance [31, 46, 48]. It has been shown that gaining access to a network of similar individuals will allow individuals to feel understood and accepted, bolstering the formation of a new ASD identity [16, 26]. A strong notion of self-acceptance can help increase psychological well-being by helping to protect against depression and anxiety [31, 48]. Participants in the current study commented on how the information gained from diagnosis allowed them to accept who they are and better plan for their future.

### Limitations and future directions

Although the current study provides unique input from the perspectives of stakeholders, this study is not without limitations. The first limitation of the current study is the lack of inquiry into the direct perspectives and experiences of adults with ASD and linking their unique symptoms to the process of late diagnosis. Additionally, all participants in the current study needed to be able to communicate verbally in English. Finally, the short time frame of the current study may not have captured the experiences of stakeholders over time.

Future studies are recommended to include a more diverse group from various geographical locations or socio-economic status to better represent the study population. Additionally, future studies should involve individuals who communicate in alternative methods as their experiences may differ from those of individuals who can communicate verbally. It is also recommended to include a multidisciplinary group of service providers in future projects to highlight their roles in diagnosis and service provision. Lastly, it is recommended to investigate individuals’ unique symptoms and experiences to capture changes in diagnostic services throughout adulthood.

## Conclusion

This study adds to the current literature regarding the lived and subjective experience of individuals receiving an ASD diagnosis during adulthood. Our findings suggest that although the impetus for seeking out diagnosis during adulthood may differ from childhood, individuals experience many of the same barriers which are exacerbated by the added challenges of adulthood diagnosis. Additionally, our findings highlight the emotional importance of receiving a diagnosis after many years of living without one and the impact it can have on individuals’ lives. Given the impact of diagnosis on individuals, it is important to minimize the barriers to ensure individuals who require ASD-related supports can access them in a timely and effective manner. The findings from the current study can be used to guide adult diagnostic processes and practices to help make ASD diagnosis more accessible.

## Data Availability

All the relevant data is available and included in the manuscript.
